# High Resolution Systematic Digital Histological Quantification of Cardiac Fibrosis and Adipose Tissue in Phospholamban p.Arg14del Mutation Associated Cardiomyopathy

**DOI:** 10.1371/journal.pone.0094820

**Published:** 2014-04-14

**Authors:** Johannes M. I. H. Gho, René van Es, Nikolas Stathonikos, Magdalena Harakalova, Wouter P. te Rijdt, Albert J. H. Suurmeijer, Jeroen F. van der Heijden, Nicolaas de Jonge, Steven A. J. Chamuleau, Roel A. de Weger, Folkert W. Asselbergs, Aryan Vink

**Affiliations:** 1 Department of Cardiology, Division Heart and Lungs, University Medical Center Utrecht, Utrecht, the Netherlands; 2 Department of Pathology, University Medical Center Utrecht, Utrecht, the Netherlands; 3 Department of Cardiology, University Medical Center Groningen, University of Groningen, Groningen, the Netherlands; 4 Department of Pathology, University Medical Center Groningen, University of Groningen, Groningen, the Netherlands; 5 Durrer Center for Cardiogenetic Research, ICIN-Netherlands Heart Institute, Utrecht, the Netherlands; 6 Institute of Cardiovascular Science, Faculty of Population Health Sciences, University College London, London, United Kingdom; Temple University, United States of America

## Abstract

Myocardial fibrosis can lead to heart failure and act as a substrate for cardiac arrhythmias. In dilated cardiomyopathy diffuse interstitial reactive fibrosis can be observed, whereas arrhythmogenic cardiomyopathy is characterized by fibrofatty replacement in predominantly the right ventricle. The p.Arg14del mutation in the phospholamban (*PLN*) gene has been associated with dilated cardiomyopathy and recently also with arrhythmogenic cardiomyopathy. Aim of the present study is to determine the exact pattern of fibrosis and fatty replacement in *PLN* p.Arg14del mutation positive patients, with a novel method for high resolution systematic digital histological quantification of fibrosis and fatty tissue in cardiac tissue. Transversal mid-ventricular slices (n = 8) from whole hearts were collected from patients with the *PLN* p.Arg14del mutation (age 48±16 years; 4 (50%) male). An in-house developed open source MATLAB script was used for digital analysis of Masson's trichrome stained slides (http://sourceforge.net/projects/fibroquant/). Slides were divided into trabecular, inner and outer compact myocardium. Per region the percentage of connective tissue, cardiomyocytes and fatty tissue was quantified. In *PLN* p.Arg14del mutation associated cardiomyopathy, myocardial fibrosis is predominantly present in the left posterolateral wall and to a lesser extent in the right ventricular wall, whereas fatty changes are more pronounced in the right ventricular wall. No difference in distribution pattern of fibrosis and adipocytes was observed between patients with a clinical predominantly dilated and arrhythmogenic cardiomyopathy phenotype. In the future, this novel method for quantifying fibrosis and fatty tissue can be used to assess cardiac fibrosis and fatty tissue in animal models and a broad range of human cardiomyopathies.

## Introduction

A network of extracellular matrix maintains the structural integrity of the myocardium. Due to several etiologies increased deposition of collagen and other extracellular matrix proteins can occur leading to cardiac fibrosis [1]. After myocardial infarction, cardiomyocytes are replaced by connective tissue leading to reparative fibrosis. In contrast, in nonischemic cardiomyopathies, an increase in collagen synthesis by myofibroblasts results in diffuse interstitial reactive fibrosis. In arrhythmogenic cardiomyopathy (AC), fibrosis is accompanied by an increase of adipocytes leading to so-called fibrofatty replacement [2].

Myocardial fibrosis is an important part of the histological characteristics in heart failure with preserved and reduced ejection fraction and may act as a substrate for cardiac arrhythmias. Adequate detection of the amount and distribution of fibrosis in the heart is important for diagnosis, predicting prognosis, treatment planning and follow-up after therapy [3], [4]. The reference noninvasive standard for indirect detection of myocardial fibrosis is late gadolinium enhancement on cardiac magnetic resonance imaging (MRI) [3]. Thus far, detailed histological correlation studies to validate this MRI technique are scarce. Histological assessment of cardiac fibrosis is mostly limited by the small amount of tissue available in diagnostic endomyocardial biopsies that only provides regional information [2]. In addition, quantification of histological fibrosis is usually performed semi-quantitatively, classifying the fibrosis in limited categories.

Phospholamban is a protein in the sarcoplasmic reticulum and acts as a (reversible) inhibitor of the Ca^2+^ pump: sarcoplasmic reticulum Ca^2+^-ATPase 2a (SERCA2a). On phosphorylation it dissociates from SERCA2a and thereby activates the Ca^2+^ pump. This cascade regulates cardiac relaxation and contractility. Several causal phospholamban (*PLN*) mutations have been described in humans [5]–[8]. The p.Arg14del (c.40_42delAGA) founder mutation in the *PLN* gene has been associated with dilated cardiomyopathy (DCM) and recently also with AC [9]. Detailed histologic analysis of the pattern of fibrosis and fatty changes in *PLN* mutation associated cardiomyopathies has not been extensively studied and to the best of our knowledge has not been performed on transverse heart slices.

The aim of this study was to determine the exact patterns of fibrosis and fatty changes in the myocardium of patients with the *PLN* p.Arg14del mutation associated cardiomyopathy in relation to their clinical phenotype. This study population was used as proof-of-principle for a novel method of high resolution systematic digital quantification of fibrosis and fatty tissue in transversal cardiac slides. In the future this method may be used for detailed histological quantification and determination of the distribution pattern of cardiac fibrosis in different types of heart disease, in addition it provides a detailed high resolution reference for imaging techniques of cardiac fibrosis.

## Methods

### Ethics statement

The study met the criteria of the code of proper use of human tissue that is used in the Netherlands. The study was approved by the scientific advisory board of the biobank of the University Medical Center Utrecht, Utrecht, the Netherlands (protocol no. 12/387). Written informed consent was obtained or in certain cases waived by the ethics committee when obtaining informed consent was not possible due to death of the patient.

Hearts obtained at autopsy (n = 2) or explantation (n = 6) were collected from patients with the *PLN* p.Arg14del mutation. Based on their initial clinical presentation, patients were divided in two categories: predominantly DCM or AC. Three control hearts, two donor hearts not-used for transplantation and one heart obtained at autopsy of a road accident victim, were used as reference.

**Figure 1:**
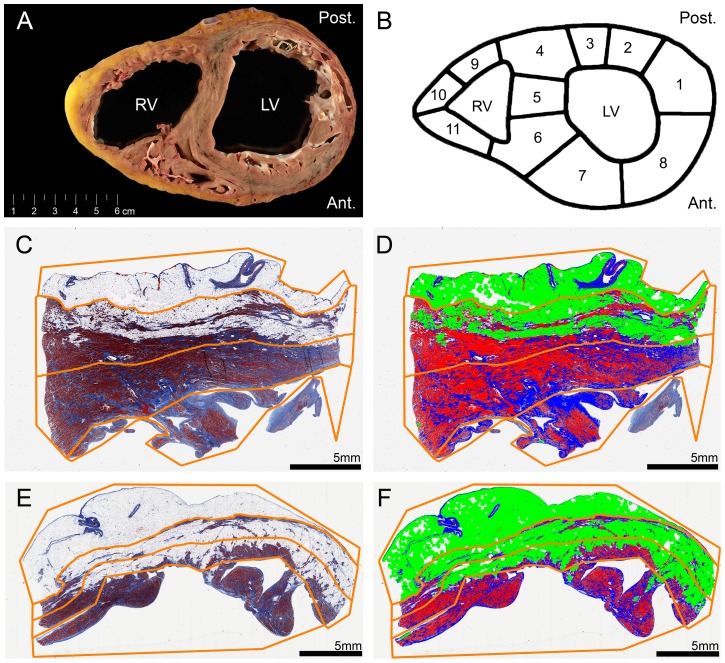
Overview of methodology. LV, left ventricle; RV, right ventricle; Ant., anterior; Post., posterior. **A,** gross showing a transverse heart slice of arrhythmogenic cardiomyopathy. **B,** transverse slice dissection scheme. **C**–**F,** examples of digital slide processing. Regions of interest are shown in orange lines (defining the epicardium, compact myocardium divided by an equidistant midline and trabeculated part). **C,** slide from the left ventricle posterior wall. **D,** slide C after digital processing. Red: cardiomyocytes. Blue: connective tissue. Pseudo green: adipose tissue. **E,** slide from the right ventricle lateral wall. **F,** slide E after digital processing. Red: cardiomyocytes. Blue: connective tissue. Pseudo green: adipose tissue.

We used a systematic methodology for high resolution digital cardiac fibrosis quantification (Fig. 1). Hearts were cut in transverse (short-axis) slices of 1 cm thick starting at the apex including both ventricles. Each fourth transverse slice was fixed in formalin and divided into smaller pieces. A map of the heart slice was drawn to annotate the origin of each tissue specimen. Subsequently the samples were embedded in paraffin and Masson's trichrome staining was performed. The slides were scanned at 20× magnification as described previously [10]. Images were extracted using Aperio ImageScope v12.0.0.5039 (Aperio, Vista, California, United States) as a TIFF file with lossless compression. The images were resized to 10% of their original size for digital analysis.

To analyze the cardiac tissue, slides were divided into several layers: the epicardial area, compact myocardium and non-compact (trabeculated) myocardium (http://sourceforge.net/projects/fibroquant/). The epicardial area was defined as the outer region of fatty tissue bordered by the first row of cardiomyocytes. The non-compact myocardial area was defined as the endocardial trabeculated region. The compact myocardium was defined by the area between the trabeculated area and the epicardial area and was artificially divided with an equidistant line in two halves. An equidistant line is one for which every point on the line is equidistant from the nearest points on both the epicardial and trabecular segmentation. In case a midline division was not feasible, e.g., due to a thin wall or originating from the interventricular septum (without epicardium), mean values of the total myocardium were used. Thus, the myocardium was divided in four layers: trabecular myocardium, inner or outer compact myocardium and the epicardium.

In all four layers the percentage of connective tissue (blue), cardiomyocytes (red) and adipose tissue (cells with non stained cytoplasm) was digitally quantified using MATLAB (Release R2012a, The MathWorks, Inc., Natick, Massachusetts, United States). The percentage per region was calculated by separating the Masson's trichrome stained slides into its constituent stains of methyl blue and ponceau-fuchsin by performing color deconvolution [11]. This produces two grayscale images depicting the concentration of the two stainings. The resulting images are filtered using a 2D median filter and further processed using morphological dilation, thresholding and closing operations. The adipose tissue is quantified using a separate function in order to highlight the “chain-link” structure of adipocytes as seen on glass slides. The original image is converted to grayscale and filtered using a 2D median filter. After that a threshold is applied and morphological opening is performed. The median filtered image is then subtracted from the morphologically opened image and the resulting image is further morphologically processed by performing consecutive closing and opening operations. This converts the “chain-link” structure into a black and white image where only the adipose tissue is left. The area of each constituent is determined and a percentage is calculated based on the total area that was processed [12]. The epicardial region was excluded from analysis. The resulting values (percentage fibrosis and fatty tissue) were annotated to the corresponding region in the heart using an automated algorithm (Fig. S1).

Subsequently the annotated map of the transverse slice is automatically transformed to a standardized schematic overview. The annotated map was translated to a schematic overview by determining the angular properties of each separate section from the middle of the ventricles with a precision of one degree. The results of the quantification (percentage fibrosis or fatty tissue) are displayed using an easily interpretable color scale. Statistics were performed using IBM SPSS Statistics (Version 20.0, IBM Corporation, Armonk, New York, United States). We compared mean percentages of fibrosis and adipose tissue in different areas between the two clinical phenotypes of AC and DCM and control hearts. The left ventricle was divided into a septal, posterior, posterolateral, lateral, anterolateral and anterior part. The right ventricle was divided into a posterior and anterior part. To compare mean percentages of fibrosis and adipose tissue corrected for surface area per region and condition a repeated measures analysis was performed. After Greenhouse-Geisser correction, interactions between region and condition were explored. Post hoc tests (Tukey HSD) were performed in the absence of a significant interaction.

## Results

**Figure 2:**
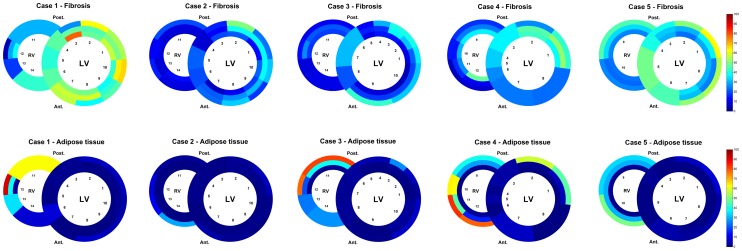
Schematic heart slice overview of patients with a clinical phenotype of dilated cardiomyopathy. The results of the digital quantification in heart slices in percentage of fibrosis or adipose tissue are shown using a color scale. The epicardial region has been excluded from this overview. LV, left ventricle; RV, right ventricle; Ant., anterior; Post., posterior

**Figure 3:**
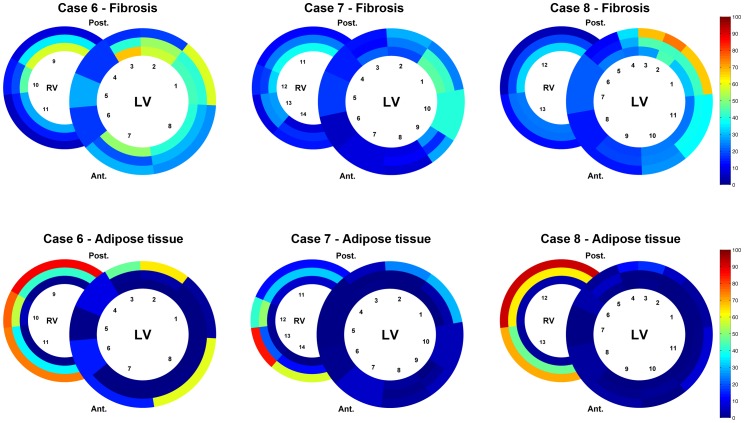
Schematic heart slice overview of patients with a clinical phenotype of arrhythmogenic cardiomyopathy. The results of the digital quantification in heart slices in percentage of fibrosis or adipose tissue are shown using a color scale. The epicardial region has been excluded from this overview. LV, left ventricle; RV, right ventricle; Ant., anterior; Post., posterior

**Figure 4:**
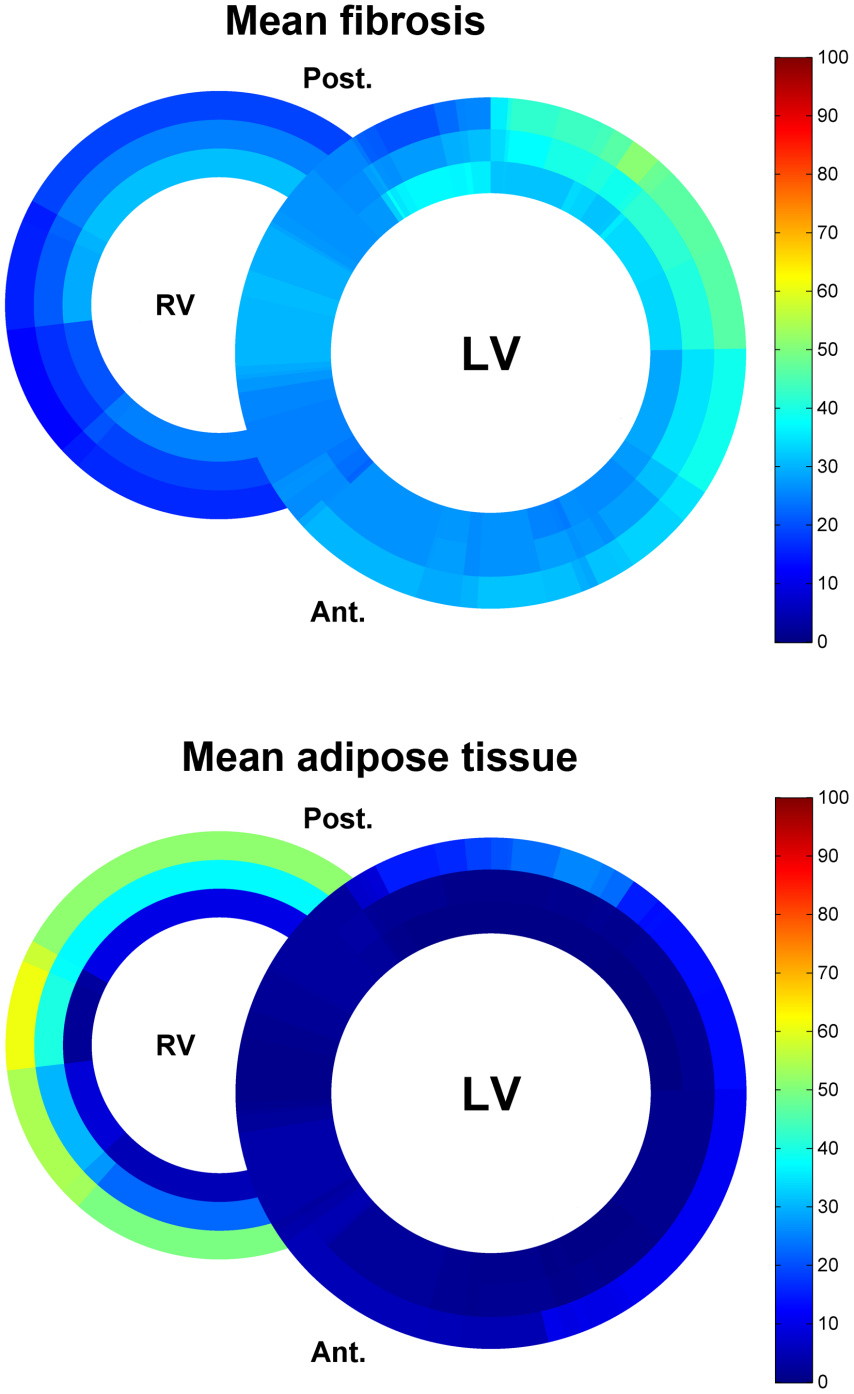
Schematic heart slice overview of patients with the *PLN* p.Arg14del mutation (dilated and arrhythmogenic cardiomyopathy). The results of the mean percentage of fibrosis or adipose tissue are shown using a color scale. In total 102 heart slides of 8 heart slices were used. LV, left ventricle; RV, right ventricle; Ant., anterior; Post., posterior

**Figure 5:**
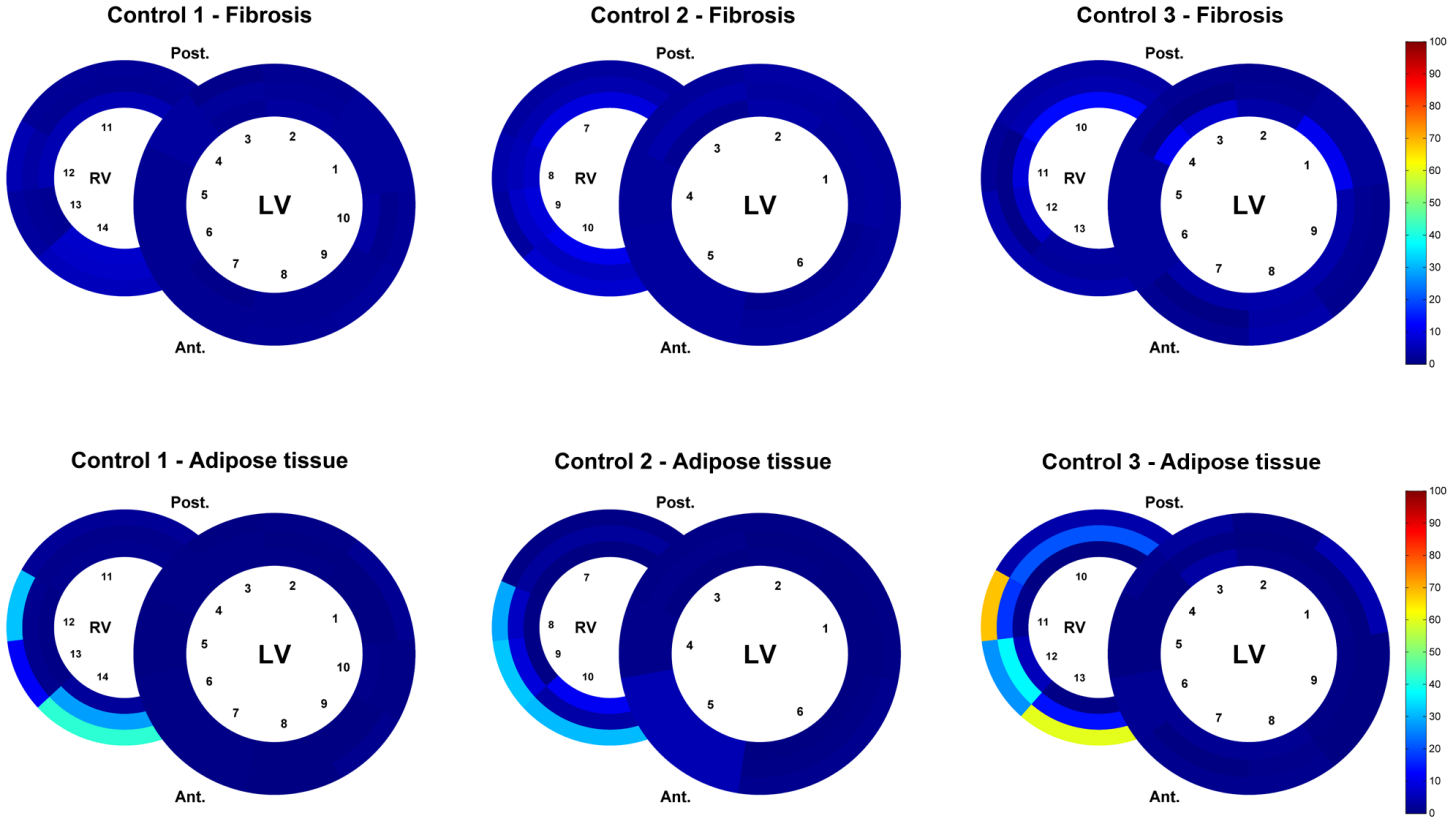
Schematic overview of fibrosis and adipose tissue in heart slices of control hearts. The results of the digital quantification in percentage of fibrosis or adipose tissue are shown using a color scale. The epicardial region has been excluded from the analysis. LV, left ventricle; RV, right ventricle; Ant., anterior; Post., posterior

Clinical characteristics of the patients are summarized in Table 1. Mean age was 48±16 years; 4 (50%) patients were male. Five patients were known with a clinical phenotype of DCM and 3 patients were known with a clinical phenotype of AC. The schematic overviews depict distribution and percentage of fibrosis and adipose tissue for DCM (Fig. 2) and arrhythmogenic patients (Fig. 3). Mean values of fibrosis and adipose tissue with standard deviations per region and condition (control, AC or DCM) are presented in Table S1. In the 8 heart slices of *PLN* mutation carriers, myocardial fibrosis was mainly observed in the trabecular part of the posterolateral wall of the right ventricle and in the posterolateral (mean >38%) and in lesser extent anterolateral (mean >26%) wall of the left ventricle. In the left ventricle, fibrosis was more pronounced in the outer layer of compact myocardium than in the myocardial layers more closely to the lumen. Mean percentage of fibrosis and adipose tissue is shown in the combined schematic overviews (Fig. 4). The septum and ventral wall of the right ventricle revealed the least amount of interstitial fibrosis. Fatty changes of myocardium were predominantly observed in the entire right ventricle wall (mean 37.2±14% and 28.9±4% in AC and 26.1±20% and 24.3±12% in DCM respectively in regions 7 and 8) and in the epicardial side of the left ventricular posterolateral wall compact myocardium (mean 6.9±5% in AC and 5.8±6% in DCM in region 1). Overall fatty infiltration of myocardium was more pronounced in the right (mean adipose tissue >24%) than in the left (mean adipose tissue <11%) ventricle. In control hearts, mean fibrosis was less than 6%, mean adipose tissue was 2% or less in the left ventricle myocardium and less than 15% in the right ventricular wall (Fig. 5).

**Table 1:** Patient characteristics of the *PLN* p.Arg14del mutation carriers.

	(n = 8)
Age; mean (SD)	48 (16)
Male sex	4 (50%)
**Device**	
ICD	4 (50%)
CRT-D	3 (38%)
Left ventricular assist device	4 (50%)
**First presenting symptom**	
Arrhythmia	4 (50%)
Heart failure	4 (50%)
Heart transplantation	6 (75%)

SD, standard deviation; ICD, implantable cardioverter-defibrillator; CRT-D, cardiac resynchronization therapy defibrillator.

**Figure 6:**
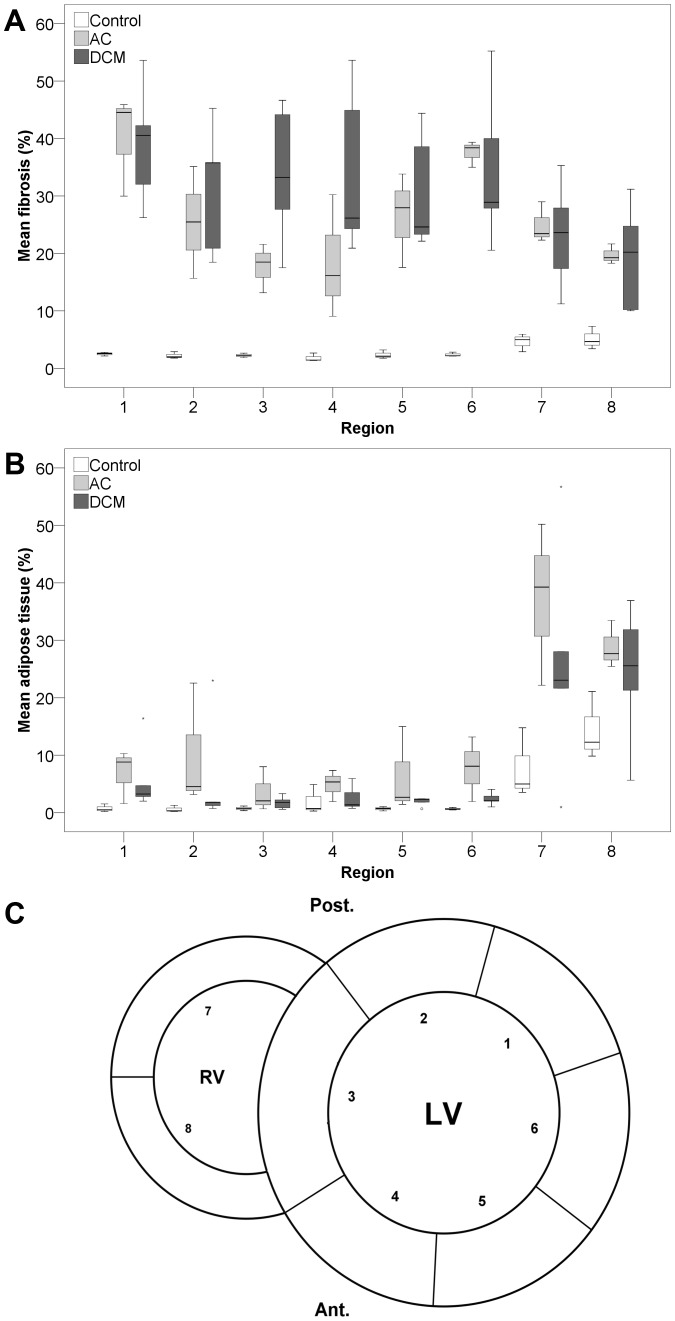
Boxplots of mean fibrosis and mean adipose tissue in the different regions. Boxplots of mean percentage of fibrosis (A) and adipose tissue (B) per condition in 8 regions (C) corrected for surface area. Outliers are represented by small circles or stars. AC, arrhythmogenic cardiomyopathy; DCM, dilated cardiomyopathy; LV, left ventricle; RV, right ventricle; Ant., anterior; Post., posterior; 1 =  LV posterolateral wall; 2  =  LV posterior wall; 3  =  interventricular septum; 4  =  LV anterior wall; 5  =  LV anterolateral wall; 6  =  LV lateral wall; 7  =  RV posterior wall; 8  =  RV anterior wall.

Mean connective and adipose tissue per condition and region (n = 8, corrected for surface area) is shown in boxplots (Fig. 6). Mean values of fibrosis and adipose tissue were log transformed to reduce right-skewness and heterogeneity of variance and a repeated measures analysis was performed. Comparing fibrosis we found a significant interaction between condition and region (p<0.001), therefore post-hoc testing was not performed. For adipose tissue there was no significant interaction between condition and region (p = 0.470). We found no significant difference between AC and DCM in pattern of adipose tissue (p = 0.382). Compared to controls, we found a higher percentage of adipose tissue in AC (p = 0.028) and a trend for a higher percentage of adipose tissue in DCM (p = 0.126).

## Discussion

To the best of our knowledge this is the first study that provides the exact and detailed pattern of fibrosis and fatty changes in *PLN* p.Arg14del mutation associated cardiomyopathy hearts. For this study we developed a novel method for systematic high resolution digital quantification of different tissue types in the heart. This quantification has been applied to Masson's trichrome stained slides of transverse cardiac slices in *PLN* p.Arg14del mutation associated cardiomyopathies. Interestingly we found an overlap in fibrosis and fatty changes between DCM and AC in *PLN* p.Arg14del mutation carriers. Myocardial fibrosis was mainly observed in the posterolateral wall of the left ventricle and in less extent in the posterolateral wall of the right ventricle. Fatty tissue was more pronounced in the myocardium bordering the epicardium of the right ventricle. We found a significant higher percentage of adipose tissue in AC compared to control hearts (p = 0.028).

Phospholamban is a regulator of the SERCA2a pump, important for maintaining Ca^2+^ homeostasis and crucial for cardiac contractility [6], [13]. Phosphorylation of PLN increases SERCA2a activity, leading to increased cardiac relaxation and contractility for the next beat. The precise pathophysiological mechanism in *PLN* p.Arg14del mutation carriers leading to cardiac fibrosis and heart failure remains unknown. Transgenic mice overexpressing the mutant PLN-R14del showed extensive myocardial fibrosis, myocyte disarray, ventricular dilation and premature death, recapitulating human cardiomyopathy [8]. Co-expression of the normal and mutant protein in HEK-293 cells resulted in SERCA2a super inhibition. From these results it was inferred that the *PLN* p.Arg14del mutation causes inhibition of the SERCA2a pump and thereby leads to disturbed calcium metabolism and subsequently cardiac dysfunction. These data show that this process of reactive fibrosis develops according to a specific pattern, irrespective of the phenotype of the patients. Our small sample size should thereby be taken into account and because of a significant interaction post-hoc testing was not feasible for comparing the mean fibrosis values. This varying phenotype might by influenced by effect modifiers, e.g., epigenetics or intense endurance exercise [14]. In addition, it has been postulated that the pattern of reactive fibrosis is determined by myocardial stress, microvascular dysfunction and sustained activation of neurohormonal and cytokine systems [15]. Future research is needed to elucidate underlying pathophysiology in *PLN* mutation carriers related to the phenotype.

Previous (not *PLN* mutation specific) histopathological studies have shown similar areas of predilection in AC [16]. At first, affected areas in the right ventricle (RV) were described as the classic triangle of dysplasia, including the RV inflow tract, the apex and the RV outflow tract [17]. Further research revealed that AC is not isolated to the RV. In a clinicopathologic study by Corrado et al. left ventricular involvement was found in 76% of cases with AC affecting both the septum and LV free wall, with a predilection for the posteroseptal and posterolateral areas [18]. A recent MRI study in desmosomal mutation positive patients with AC showed involvement of the basal inferior and anterior right ventricle and the posterolateral left ventricle in AC, supporting the presence of a new biventricular triangle in early AC [19]. Our results support the evidence from experimental animal models that the disease process in AC starts on the epicardial side and extends as a wave front from the epicardium towards the endocardium [20]. Clinical diagnosis of AC is made using International Task Force (revised) criteria, including structural (MRI and echocardiogram), histological (e.g. endomyocardial biopsy), electrocardiographic, arrhythmic and genetic features [2]. The sensitivity of endomyocardial biopsies from the right ventricular septum in AC is low, according to our findings this might also be the case in *PLN* mutation associated cardiomyopathies [21].

The method of fibrosis quantification presented here can be used for several applications. First, determination of the fibrosis pattern in the heart could provide an important link for genotype-phenotype relationships in genetic cardiomyopathies. Previous studies have proposed morphometric evaluation of either fibrosis or adipocytes in different tissues [22]–[24]. In our analysis fibrosis and fibrofatty replacement can be assessed simultaneously in layer specific detail. We defined different regions of interest to divide the heart in an epicardial, compact and trabeculated layer. In addition, the pattern of fibrosis can be studied in the different regions of both ventricles. By studying the exact fibrosis pattern throughout the heart, patterns of disease might be discovered thereby elucidating mechanisms of pathophysiology. Numerous disease-causing genes for different cardiomyopathies have been identified during the past two decades and the challenge for the future is to link these genetic mutations to specific patterns of disease in the heart [20], [25]. In future, AC with different underlying causal mutations could be compared to the *PLN* p.Arg14del mutation carriers with AC.

The second application could be validation of cardiac imaging techniques. The reference noninvasive standard for fibrosis detection is late gadolinium enhancement on MRI. [3] Adequate correlation to the gold standard of histology is important. Thus far, correlation studies are mostly done with small endomyocardial biopsies that only represent a fraction of the total myocardium or with triphenyl tetrazolium chloride (TTC) stained heart slices [26]. Recently, several novel techniques for fibrosis detection have been proposed, including T_1_-mapping, that also require adequate correlation to histology [3], [27]. Cardiac MRI images obtained before autopsy or heart transplantation, indicating fibrosis could be divided in similar segments to produce a bull's eye plot for comparison with histopathological quantification. However, some heart failure patients are ineligible for MRI because of implanted devices, such as implantable cardioverter-defibrillator, cardiac resynchronization therapy and left ventricular assist devices.

A third potential application could be systematic fibrosis quantification in animal models, for example in models of ischemic [28] or nonischemic cardiomyopathy [29]. Effects of novel therapies on myocardial fibrosis can be examined in randomized preclinical trials, by systematically comparing the amount of fibrosis on histology. A standardized preclinical model with induced myocardial infarction can be used to study new therapeutics, such as cell therapy, as a strategy to attenuate cardiac fibrosis and stop progression towards heart failure [30], [31].

In conclusion, in *PLN* p.Arg14del mutation associated cardiomyopathy myocardial fibrosis is predominantly present in the left posterolateral wall, whereas fatty changes are more pronounced in the wall of the right ventricle. In the analyzed heart slices from *PLN* p.Arg14del mutation carriers with nonischemic cardiomyopathy we found an overlap in distribution pattern between patients with DCM and AC and a significant higher percentage of adipose tissue in AC compared to control hearts (p = 0.028). We developed a novel method for systematic high resolution digital histological quantification of fibrosis and fatty tissue in the heart. This method can be used to assess cardiac fibrosis and fatty tissue in a broad range of human cardiomyopathies, animal models and can serve as gold standard for noninvasive imaging techniques.

## Supporting Information

Figure S1 **Schematic overview of Case 6 Arrhythmogenic Cardiomyopathy with representative images. A,B,** schematic heart slice overview of mean fibrosis (A) and adipose tissue (B). Corresponding slide numbers are shown inside the inner rings (n = 11). Mean percentage of fibrosis (A) or adipose tissue (B) per area have been superimposed on the overview (values rounded to the nearest whole number, values <20% are shown in white to improve readability). **C,E,G,** raw microscopic slide images after Masson's trichrome staining, respectively corresponding to slide 1, 5 and 7. **D,F,H,** corresponding slides after digital processing. Red: cardiomyocytes. Blue: connective tissue. Pseudo green: adipose tissue. The slides depicted in **Figure 1C-F** are also derived from the same heart, **1C,D** correspond to slide 3 and **1E,F** correspond to slide 10.
(TIF)

Table S1 **Mean percentage of fibrosis and adipose tissue per region and condition.** Regions correspond to the depicted regions in **Figure 6C**. AC, Arrhythmogenic Cardiomyopathy; DCM, Dilated Cardiomyopathy; SD, standard deviation.
(DOCX)
