# Supplementary figures and images for: High Resolution Systematic Digital Histological Quantification of Cardiac Fibrosis and Adipose Tissue in Phospholamban p.Arg14del Mutation Associated Cardiomyopathy
Source: PLoS One. 2014 Apr 14;9(4):e94820. doi: 10.1371/journal.pone.0094820 (PMC3986391; doi:10.1371/journal.pone.0094820)

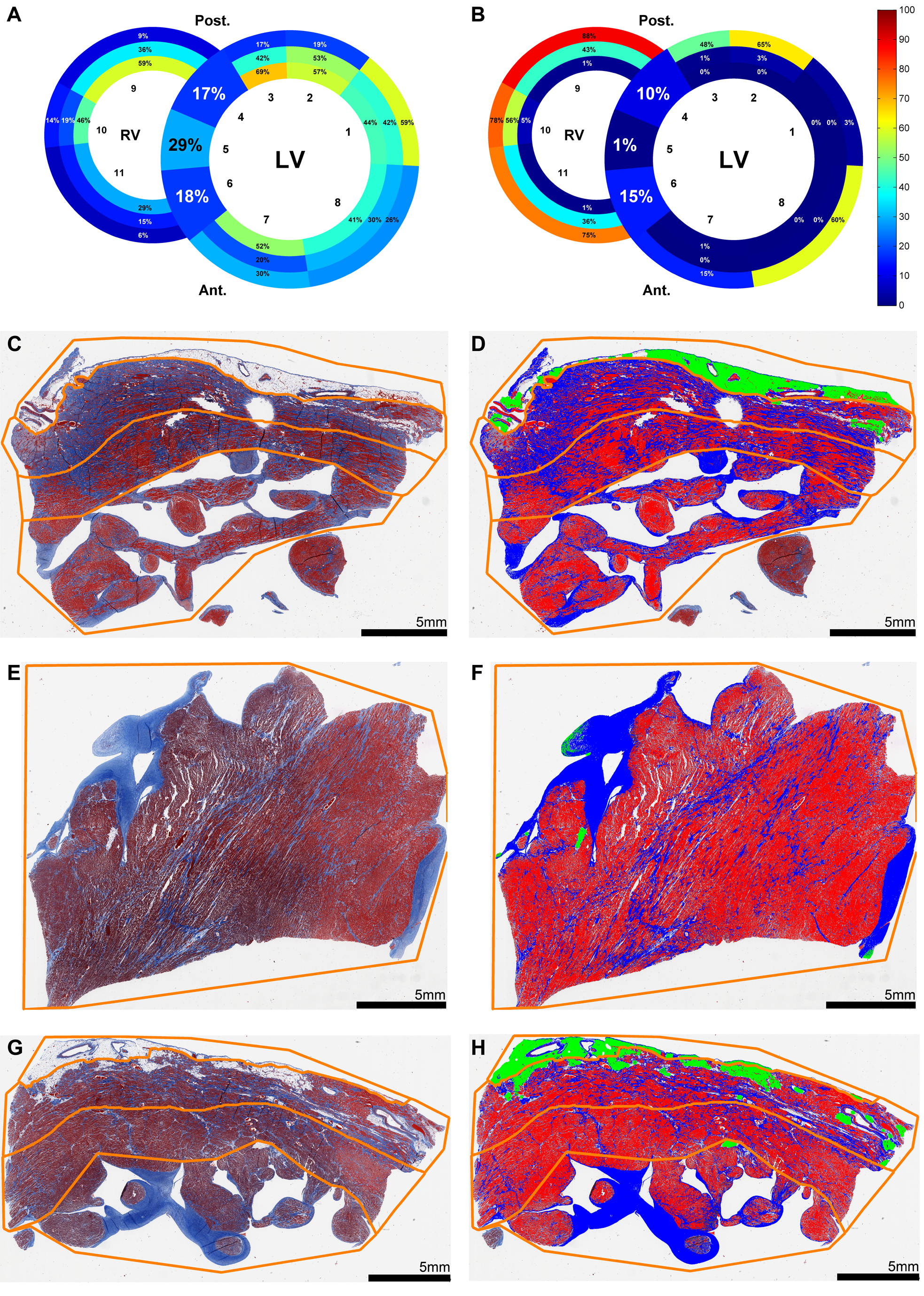

Supplement: Figure S1 — Schematic overview of Case 6 Arrhythmogenic Cardiomyopathy with representative images. A,B, schematic heart slice overview of mean fibrosis (A) and adipose tissue (B). Corresponding slide numbers are shown inside the inner rings (n = 11). Mean percentage of fibrosis (A) or adipose tissue (B) per area have been superimposed on the overview (values rounded to the nearest whole number, values <20% are shown in white to improve readability). C,E,G, raw microscopic slide images after Masson's trichrome staining, respectively corresponding to slide 1, 5 and 7. D,F,H, corresponding slides after digital processing. Red: cardiomyocytes. Blue: connective tissue. Pseudo green: adipose tissue. The slides depicted in Figure 1C-F are also derived from the same heart, 1C,D correspond to slide 3 and 1E,F correspond to slide 10. (TIF) [file pone.0094820.s001.tif]
