# Supplementary material for: High Resolution Systematic Digital Histological Quantification of Cardiac Fibrosis and Adipose Tissue in Phospholamban p.Arg14del Mutation Associated Cardiomyopathy
Source: PLoS One. 2014 Apr 14;9(4):e94820. doi: 10.1371/journal.pone.0094820 (PMC3986391; doi:10.1371/journal.pone.0094820)
Supplement: Table S1 — Mean percentage of fibrosis and adipose tissue per region and condition. Regions correspond to the depicted regions in Figure 6C . AC, Arrhythmogenic Cardiomyopathy; DCM, Dilated Cardiomyopathy; SD, standard deviation. (DOCX) [file pone.0094820.s002.docx]

**Table S1 - Mean percentage of fibrosis and adipose tissue per region and condition**

|  |  | Control (n=3) | | AC (n=3) | | DCM (n=5) | |
| --- | --- | --- | --- | --- | --- | --- | --- |
|  |  | Mean (%) | SD | Mean (%) | SD | Mean (%) | SD |
| Fibrosis | Region 1 | 2.5 | 0.3 | 40.1 | 8.8 | 38.9 | 10.5 |
|  | Region 2 | 2.2 | 0.6 | 25.4 | 9.7 | 31.2 | 11.3 |
|  | Region 3 | 2.3 | 0.4 | 17.7 | 4.3 | 33.8 | 12.0 |
|  | Region 4 | 1.8 | 0.7 | 18.5 | 10.8 | 34.0 | 14.4 |
|  | Region 5 | 2.4 | 0.8 | 26.4 | 8.2 | 30.6 | 10.2 |
|  | Region 6 | 2.4 | 0.4 | 37.6 | 2.3 | 34.5 | 13.5 |
|  | Region 7 | 4.6 | 1.6 | 24.9 | 3.6 | 23.1 | 9.3 |
|  | Region 8 | 5.1 | 2.0 | 19.7 | 1.7 | 19.3 | 9.2 |
| Adipose tissue | Region 1 | 0.7 | 0.7 | 6.9 | 4.7 | 5.8 | 6.0 |
|  | Region 2 | 0.6 | 0.6 | 10.1 | 10.8 | 5.7 | 9.7 |
|  | Region 3 | 0.7 | 0.4 | 3.6 | 3.9 | 1.8 | 1.1 |
|  | Region 4 | 2.0 | 2.5 | 4.9 | 2.7 | 2.5 | 2.2 |
|  | Region 5 | 0.7 | 0.4 | 6.4 | 7.5 | 1.9 | 0.7 |
|  | Region 6 | 0.7 | 0.2 | 7.7 | 5.6 | 2.4 | 1.1 |
|  | Region 7 | 7.8 | 6.1 | 37.2 | 14.1 | 26.1 | 20.0 |
|  | Region 8 | 14.4 | 5.9 | 28.9 | 4.1 | 24.3 | 12.0 |
